# Effects of the polymorphisms rs17782313 in mc4r gene and rs2282679 in VDBP gene on the TG-HDL ratio and HOMA-IR through pregnancy

**DOI:** 10.1186/1758-5996-7-S1-A223

**Published:** 2015-11-11

**Authors:** Nelly Janet Trujillo Bagnasco, Adriana Rita Schultz Moreira, Maisa Martins, Dayana Farias, Gilberto Kac

**Affiliations:** 1 UFRJ, Rio de Janeiro, Brazil

## Background

Maternal adaptations during pregnancy are needed to support the adequate growth and development of the fetus, delivery and lactation. They generate an insulin resistance status and altered lipid metabolism, which can evolve into an imbalance that may precede diabetes and cardiovascular diseases. These patterns of alterations are a result of different interaction amongst genes and environmental factors.

## Objective

To evaluate the effects of polymorphisms rs17782313 in mc4r gene and rs2282679 in vdbp gene on the TG-HDL ratio and HOMA-IR through pregnancy.

## Materials and methods

146 pregnant women from a prospective cohort study conducted at a prenatal unit in Rio de Janeiro, Brazil, from November 2009 to July 2012 were evaluated. Eligibility criteria were: ≤13 gestational weeks, between 20th to 40th yrs. old and no infectious or chronic diseases (except obesity). Women were followed-up in the gestational weeks: 5-13th, 20-26th and 30-36th. Gestational diabetes diagnosis was self-reported. Triglycerides (TG), HDL-cholesterol (HDL), insulin and glucose levels were measured from blood samples collected after 12-hour fasting. HOMA-IR and TG-HDL ratio were calculated. DNA was extracted from blood samples by phenol-chloroform method. Participants were genotyped for the polymorphisms rs17782313 in melanocortin 4 receptor (mc4r) gene and rs2282679 in vitamin D binding protein (vdbp) gene by Real Time PCR method. They were analyzed as carriers CC/CT (homocygotes CC and heterocygotes CT for the rs17782313) and GG/GT (homocygotes GG and heterocygotes GT for the rs2282679) against non-carriers. We evaluated differences for variables between groups by using the Mann-Whitney test.

## Results

Mean (sd) maternal age (yrs.) was 27.0 (5.5) and there were 12% women with BMI ≥30 kg/m2 at early pregnancy and 7% with self-reported gestational diabetes. Frequencies of carriers CC/CT and GG/GT were 34.5% and 33.6%, respectively. Women with CC/CT showed significant difference of TG-HDL ratio against non-carriers at early pregnancy (p=0.02). For the 20-26th and 30-36th gestational weeks, carriers GG/GT had significant differences of TG-HDL ratio when compared to non-carriers (p <0.05). For HOMA-IR, differences were not found for both polymorphisms in any pregnancy period.

## Conclusion

The vdbp polymorphism seems to be more related to the lipid metabolism than the insulin resistance, perhaps it can be mediated by the interaction between dietary intake and polymorphism.

